# Thiol-Reactive PODS-Bearing Bifunctional Chelators for the Development of EGFR-Targeting [^18^F]AlF-Affibody Conjugates

**DOI:** 10.3390/molecules25071562

**Published:** 2020-03-29

**Authors:** Chiara Da Pieve, Ata Makarem, Stephen Turnock, Justyna Maczynska, Graham Smith, Gabriela Kramer-Marek

**Affiliations:** 1 PET Radiochemistry, Division of Radiotherapy and Imaging, the Institute of Cancer Research, 123 Old Brompton Road, London SW7 3RP, UK; Graham.Smith@icr.ac.uk; 2 Division of Radiopharmaceutical Chemistry, German Cancer Research Center (DKFZ), Im Neuenheimer Feld 280, 69120 Heidelberg, Germany; a.makarem@dkfz-heidelberg.de; 3 Preclinical Molecular Imaging, Division of Radiotherapy and Imaging, the Institute of Cancer Research, 123 Old Brompton Road, London SW7 3RP, UK; Stephen.Turnock@icr.ac.uk (S.T.); justyna.maczynska@icr.ac.uk (J.M.)

**Keywords:** [^18^F]AlF, NOTA, NODAGA, PODS, thiol-reactive, linker, affibody molecule, bioconjugation, EGFR, tumor imaging

## Abstract

Site-selective bioconjugation of cysteine-containing peptides and proteins is currently achieved via a maleimide–thiol reaction (Michael addition). When maleimide-functionalized chelators are used and the resulting bioconjugates are subsequently radiolabeled, instability has been observed both during radiosynthesis and post-injection in vivo, reducing radiochemical yield and negatively impacting performance. Recently, a phenyloxadiazolyl methylsulfone derivative (PODS) was proposed as an alternative to maleimide for the site-selective conjugation and radiolabeling of proteins, demonstrating improved in vitro stability and in vivo performance. Therefore, we have synthesized two novel PODS-bearing bifunctional chelators (NOTA-PODS and NODAGA-PODS) and attached them to the EGFR-targeting affibody molecule Z_EGFR:03115_. After radiolabeling with the aluminum fluoride complex ([^18^F]AlF), both conjugates showed good stability in murine serum. When injected in high EGFR-expressing tumor-bearing mice, [^18^F]AlF-NOTA-PODS-Z_EGFR:03115_ and [^18^F]AlF-NODAGA-PODS-Z_EGFR:03115_ showed similar pharmacokinetics and a specific tumor uptake of 14.1 ± 5.3% and 16.7 ± 4.5% ID/g at 1 h post-injection, respectively. The current results are encouraging for using PODS as an alternative to maleimide-based thiol-selective bioconjugation reactions.

## 1. Introduction

Reactive sulfhydryl groups of cysteine residues are attractive sites for the chemical attachment of dyes, chelators, or drugs to biomolecules. Targeting cysteine residues on biomolecules has several key benefits. Firstly, the presence of the thiol group, a highly reactive nucleophile, allows for a fast and selective reaction at physiological pH. The natural low abundance of accessible and reduced cysteine residues prevents the formation of heterogeneous mixtures of the bioconjugates. Moreover, a customized site-specific incorporation of cysteine residues into a biomolecule can be easily achieved [1]. Different classes of thiol-targeting electrophilic compounds have been used, with maleimides being the most common choice [2,3,4]. However, maleimide conjugates have shown instability mostly as a consequence of thiol exchange reactions in vivo (e.g., retro-Michael addition) [5,6]. When maleimide-based bioconjugates are used as imaging agents, the above mentioned succinimidyl thioether linkage instability can lead to reduced accumulation in target tissues due to the release of the radioactive payload from the conjugate. Additionally, the thiol exchange reaction with endogenous thiol-containing biomolecules (e.g., albumin, cysteine and glutathione) can result in a higher background-to-noise ratio and a consequently reduced imaging contrast [7]. Thiol-reactive reagents have been investigated aiming at the formation of selective, fast, high-yielding and, most importantly, stable linkages with biomolecules [2,8,9,10]. Among the promising molecules, oxadiazolyl methyl sulfone-based compounds showed not only rapid and selective reaction with thiols in proteins but also the capacity of forming conjugates (via an oxadiazole–protein thiolate bond) which were more stable than those derived from maleimides [11,12]. The preparation of thiol-reactive bifunctional chelators and prosthetic groups containing the oxadiazolyl methylsulfone moiety (e.g., phenyloxadiazolyl methylsulfone or PODS) and their attachment to proteins and peptides have been examined [13,14,15]. Once radiolabeled, the conjugates were found to be more stable in vitro than the maleimide-derived counterparts. Moreover, when used in vivo, the oxadiazolyl methylsulfone linker-derived agents demonstrated reduced uptake in non-targeted tissues than the maleimide equivalents [13,14,15]. Based on these promising reports, we have prepared two novel phenyloxadiazolyl methylsulfone-containing (PODS) bifunctional chelators (NOTA-PODS and NODAGA-PODS) for the conjugation and aluminum fluoride ([^18^F]AlF) radiolabeling of a cysteine-containing biomolecule. For our study, an EGFR-targeting affibody molecule (Z_EGFR:03115_) was used as a thiol-bearing protein representative. The effect of the chelator structure on the synthesis and radiolabeling of the two conjugates ([^18^F]AlF-NOTA-PODS-Z_EGFR:03115_ and [^18^F]AlF-NODAGA-PODS-Z_EGFR:03115_) was studied together with their in vitro and in vivo profile. Additionally, a comparison was carried out with the maleimide-bearing [^18^F]AlF-NOTA-Z_EGFR:03115_ to benchmark in vitro and in vivo performance.

## 2. Results and Discussion

### 2.1. Preparation of Bifunctional Chelators NOTA-PODS and NODAGA-PODS

**Scheme 1:**
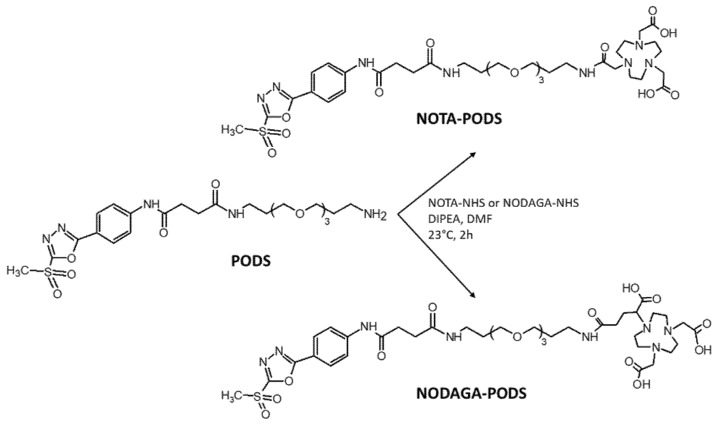
Synthesis of NOTA-PODS and NODAGA-PODS.

The aluminum fluoride-18 ([^18^F]AlF) radiolabeling procedure has generated significant interest by combining the convenient and straightforward metal-based radiochemistry (e.g., one-pot reaction, minimal purification steps, aqueous solution compatibility) and the excellent decay characteristics of fluorine-18 (97% positron emission, low positron energy, half-life of 109.8 min). This method was used to directly radiolabel small molecules, peptides and proteins containing macrocyclic chelators such as NOTA or NODA [16,17]. Accordingly, in this work, the NOTA and NODAGA chelators were selected for the [^18^F]AlF radiolabeling of affibody molecule Z_EGFR:03115_. However, instead of the conventional maleimide, thiol-reactive bifunctional chelators for the conjugation to the cysteine-containing small protein were developed by functionalizing the macrocycles with PODS. The reaction between PODS and the NHS ester of either NOTA or NODAGA was performed in DMF in the presence of base (Scheme 1). The products were obtained in a quantitative yield (Figure S1); NOTA-PODS and NODAGA-PODS were used for the subsequent conjugation reaction without further purification. Alternatively, pure bifunctional chelators could be isolated by semi-preparative RP–HPLC using formic acid instead of TFA in the mobile phase (Figures S2–S4). Of note, when NOTA-PODS solutions in DMF were stored at −20 °C, noticeable signs of degradation were detected already after 2 months. Conversely, NODAGA-PODS in DMF showed good stability for at least 10 months (Figure S5).

### 2.2. Preparation of NOTA-PODS-Z_EGFR:03115_ and NODAGA-PODS-Z_EGFR:03115_

**Scheme 2:**
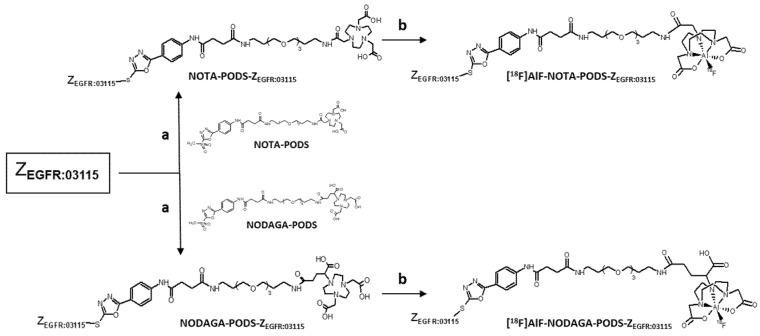
Preparation of [^18^F]AlF-NOTA-PODS-Z_EGFR:03115_ and [^18^F]AlF-NODAGA-PODS-Z_EGFR:03115_. Reaction conditions: (a) TCEP-HCl, 1 M phosphate buffer pH 7; (b) AlCl_3_ in sodium acetate pH 4, [^18^F]F^-^ aq./ethanol 1:1 (*v*/*v*), for 15 min, and at 100 °C.

**Figure 1:**
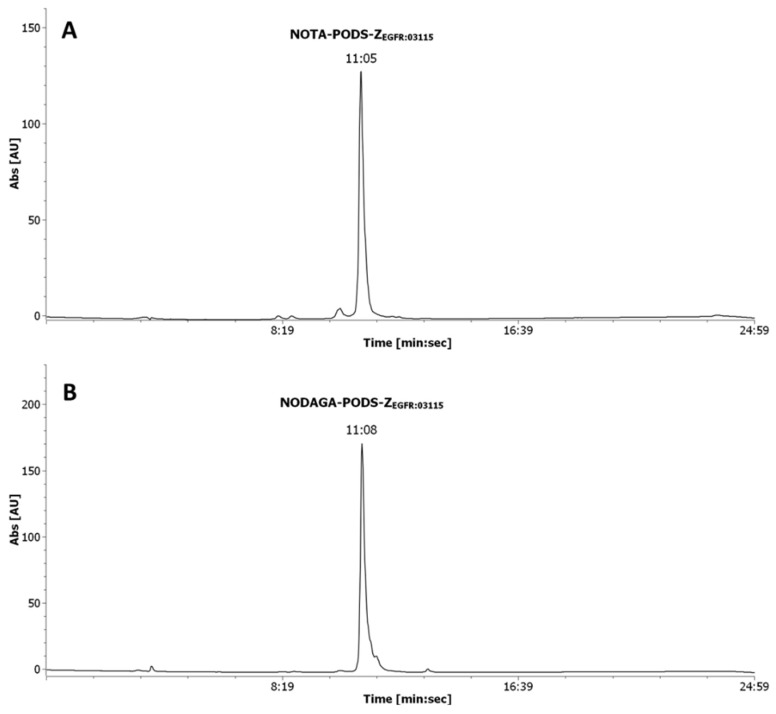
RP–HPLC analysis of NOTA-PODS-Z_EGFR:03115_ (**A**), and NODAGA-PODS-Z_EGFR:03115_ (**B**). The absorbance was recorded at the wavelength of 280 nm. The retention time (Rt) is indicated as min:sec.

The bifunctional chelators were conjugated to Z_EGFR:03115_ in a one-pot reaction using TCEP-HCl as the reducing agent (Scheme 2, Figure S6). A quantitative yield was achieved using a lower molar excess of PODS-bifunctional chelator to protein compared to a conventional maleimide-based chelator (15 vs. 35 equivalents). Being the great molar excess necessary as a consequence of the inhibition of the reactivity of maleimide-based compounds by TCEP, this result suggests that PODS is not as susceptible to TCEP as maleimides [18]. To obtain extremely pure products for the subsequent radiolabeling reaction, a single final purification of the conjugates by semi-preparative HPLC was performed (Figure 1). The pure compounds were isolated in a ca. 38% and ca. 24% yield for NOTA-PODS-Z_EGFR:03115_ and NODAGA-PODS-Z_EGFR:03115_, respectively. All products were characterized by ESI mass spectrometry (Figure S7).

### 2.3. [^18^F]AlF Radiolabeling and In Vitro Stability

**Figure 2:**
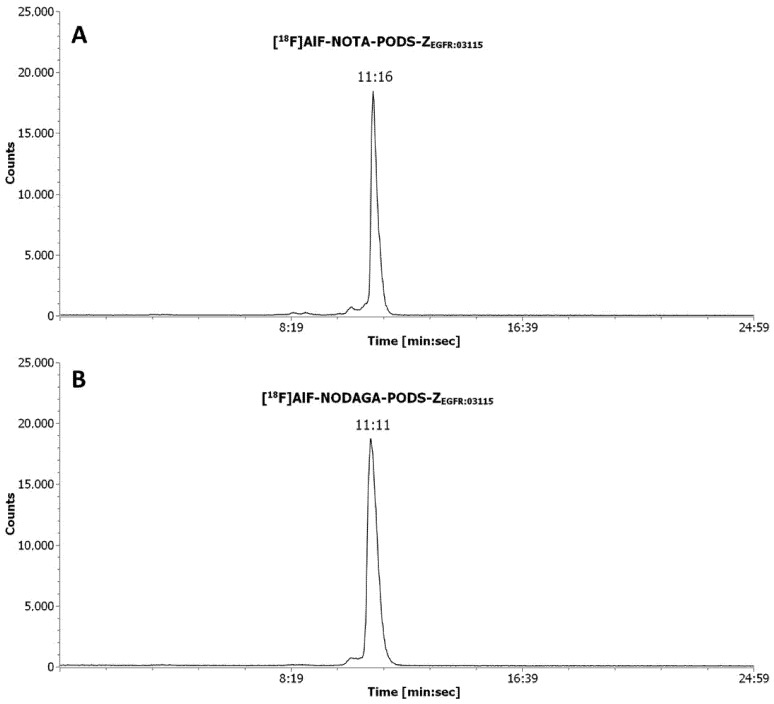
Radiochromatograms of pure [^18^F]AlF-NOTA-PODS-Z_EGFR:03115_ (**A**), and [^18^F]AlF-NODAGA-PODS-Z_EGFR:03115_ (**B**). The retention time (Rt) is indicated as min:sec.

The affibody conjugates were radiolabeled with [^18^F]AlF in a one-pot reaction at pH 4, 100 °C for 15 min using ethanol as organic co-solvent (50% *v*/*v*) (Scheme 2). Consistent with previously reported results for maleimide-derived affibody conjugates, some thermal degradation products were formed during the reaction (Figures S8,S9) [17]. Therefore, a RP–HPLC purification procedure, followed by HLB-SPE, was required to produce [^18^F]AlF-NOTA-PODS-Z_EGFR:03115_ and [^18^F]AlF-NODAGA-PODS-Z_EGFR:03115_ with a RCP > 98%, verified by RP–HPLC (Figure 2). As shown in Table 1, the radiochemical yields and the apparent specific/molar activities (SA/MA) achieved for [^18^F]AlF-NODAGA-PODS-Z_EGFR:03115_ were lower than for [^18^F]AlF-NOTA-PODS-Z_EGFR:03115_. Similar findings have been described by other research groups and attributed to the chelator structure and chelation capacity towards the [^18^F]AlF complex [19,20].

To investigate the impact of the PODS linker on the radioconjugate properties, the distribution coefficient (logD_7.4_) values of the two products were determined (Table S1). The logD_7.4_ for [^18^F]AlF-NOTA-PODS-Z_EGFR:03115_ and [^18^F]AlF-NODAGA-PODS-Z_EGFR:03115_ was found to be hydrophilic, with values of −1.73 ± 0.07 and −3.62 ± 0.06, respectively. Including the maleimide-bearing product [^18^F]AlF-NOTA-Z_EGFR:03115_ logD_7.4_ value of −1.13 ± 0.1, the NODAGA-radioconjugate proved to be the most hydrophilic of the tested compounds.

The stability of the PODS-bearing radioconjugates, with respect to change in radiochemical purity (RCP) and loss of radioactivity from the affibody molecule, was determined in mouse serum at 37 °C for 1 h (Table S2). As affibody molecules have short blood half-life, and the in vivo studies (imaging and biodistribution) are typically performed 1 h post-injection, a longer incubation time was not investigated [21]. According to RP–HPLC analysis, both conjugates exhibited good stability as 92.7 ± 2.6% of [^18^F]AlF-NOTA-PODS-Z_EGFR:03115_ and 97.2 ± 1.2% of [^18^F]AlF-NODAGA-PODS-Z_EGFR:03115_ remained intact after 1 h (Figure S10, Table S2). As previously observed for [^18^F]AlF-NOTA-Z_EGFR:03115_, a residual activity associated with the pelleted protein indicates some non-specific affinity of the radioconjugates towards the serum proteins (Table S2). However, amongst the tested compounds, the maleimide-bearing [^18^F]AlF-NOTA-Z_EGFR:03115_ demonstrated significantly higher protein-associated radioactivity than [^18^F]AlF-NOTA-PODS-Z_EGFR:03115_ and [^18^F]AlF-NODAGA-PODS-Z_EGFR:03115_ (30.5 ± 2.1% vs. 24.2 ± 3.4% and 20.4 ± 0.7%, respectively). Studies to determine whether this effect is connected to the maleimide linker are under investigation.

### 2.4. In Vivo Studies

**Figure 3:**
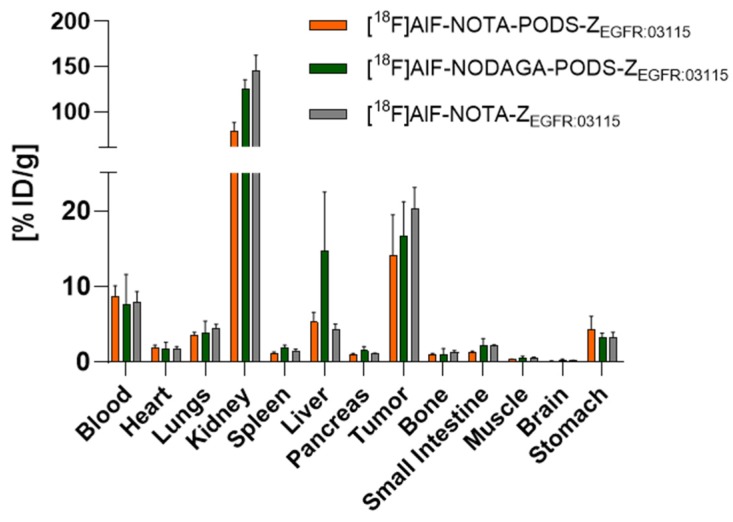
Biodistribution results for [^18^F]AlF-NOTA-PODS-Z_EGFR:03115_ and [^18^F]AlF-NODAGA-PODS-Z_EGFR:03115_ at 1 h p.i. [^18^F]AlF-NOTA-Z_EGFR:03115_ was used as a control. The data are reported as the mean percentage of the injected dose per gram of tissue (%ID/g) ± SD (for each group, *n* = 3).

**Figure 4:**
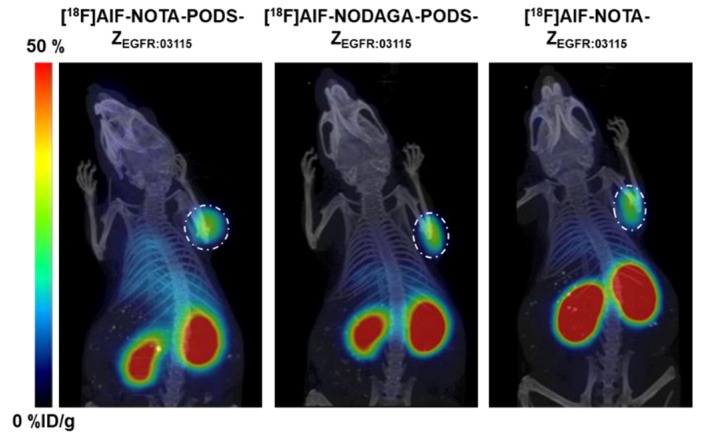
PET/CT images of U87MGvIII tumor-bearing mice using [^18^F]AlF-NOTA-PODS-Z_EGFR:03115_ and [^18^F]AlF-NODAGA-PODS-Z_EGFR:03115_. [^18^F]AlF-NOTA-Z_EGFR:03115_ was used as control. High-contrast images were acquired as early as 1 h p.i. The tumors are indicated by a white circle.

To investigate the chelator effect on the pharmacokinetics and targeting properties of the conjugates, the two PODS-bearing radioactive agents were assessed in vivo in high EGFR-expressing U87MGvIII tumor-bearing mice. To determine the influence of PODS, a comparison to the maleimide derivative [^18^F]AlF-NOTA-Z_EGFR:03115_ was performed. Previous studies found that 12 µg of radiolabeled EGFR-targeting affibody conjugate with an apparent specific activity of 0.09–0.15 MBq/µg was able to partially saturate the endogenous EGFR expression (e.g., liver) and allowed for clear visualization of the tumors 1 h post-injection [22]; therefore, a dose of 12 µg (1.1–1.8 MBq) of each radioconjugate was injected. The biodistribution data indicate that the nature of the linker (i.e., PODS or maleimide) minimally influenced the radioconjugate pharmacokinetics, since similar distribution profiles were observed by both the NOTA-based products [^18^F]AlF-NOTA-PODS-Z_EGFR:03115_ and [^18^F]AlF-NOTA-Z_EGFR:03115_ (Figure 3, Table S3). However, compared to the NOTA-bearing products, [^18^F]AlF-NODAGA-PODS-Z_EGFR:03115_ showed an inconsistent radioactivity accumulation in the liver. This result suggests that the chelator can influence the radioconjugate pharmacokinetics and that chelator-customized injected protein doses and specific activities should be investigated further by dose-escalation studies. Among the non-targeted organs, the highest uptake of all three conjugates was found in the kidneys, which is due to renal excretion of the affibody molecule, and the reabsorption of the radioactive metabolites followed by their retention in the proximal tubular cells [23]. Based on this effect, the kidney uptake value for [^18^F]AlF-NODAGA-PODS-Z_EGFR:03115_ suggests a possible higher renal clearance of the NODAGA conjugate compared to [^18^F]AlF-NOTA-PODS-Z_EGFR:03115_. Moreover, both PODS-bearing radioconjugates show significantly lower kidney uptakes than the maleimide analogue, indicating a possible effect of the PODS linker on the renal retention (Figure 3 and Table S3). More experiments will be needed to confirm this observation. High accumulation of the conjugates was found in the tumors, with uptakes of 14.1 ± 5.3 and 16.7 ± 4.5 %ID/g for [^18^F]AlF-NOTA-PODS-Z_EGFR:03115_ and [^18^F]AlF-NODAGA-PODS-Z_EGFR:03115_, respectively (Figure 4, Table S3). The favorable tumor-to-muscle ratios for the two PODS-bearing radioconjugates resulted in high-contrast PET images already 1 h post-injection (Table 2). In contrast to Adumeau et al., there was no difference in both the biodistribution profile and the tumor-to-tissue ratios between the [^18^F]AlF PODS-bearing conjugates and the malemide-based product (Table 2, Table S3) [15].

## 3. Materials and Methods

### 3.1. General Materials and Methods

Chemicals and solvents were purchased and used without further purification unless otherwise stated. The 2,2′-(7-{2-[(2,5-dioxopyrrolidin-1-yl)oxy]-2-oxoethyl}-1,4,7-triazonane-1,4-diyl)diacetic acid (NOTA-NHS) and 2,2′-(7-{1-carboxy-4-[(2,5-dioxopyrrolidin-1-yl)oxy)-4-oxobutyl]-1,4,7-triazonane-1,4-diyl}diacetic acid (NODAGA-NHS) were purchased from Chematech (Dijon, France). Tris(2-carboxyethyl)-phosphine hydrochloride (TCEP-HCl) was purchased from Thermo Fisher Scientific (Loughborough, UK). Ethanol (EtOH) and HPLC grade acetonitrile, trifluoroacetic acid (TFA) and formic acid were purchased from Thermo Fisher Scientific (Loughborough, UK). Phosphate-buffered saline (PBS) was purchased from Gibco (Life Technologies, Paisley, UK). Ethylenediaminetetraacetic acid (EDTA), *N*,*N*-diisopropylethylamine (DIPEA), mouse serum, dimethylformamide (DMF), *n*-octanol and Iso-Disc PVDF syringe filters (13 mm, 0.2 µm) were purchased from Sigma-Aldrich (Gillingham, UK). Aluminum chloride hexahydrate (AlCl_3_, 99.9995%) was purchased from Alfa Aesar (Heysham, UK). Sodium acetate (AnalR Normapur) was purchased from VWR International (Lutterworth, UK). The Z_EGFR:03115_-Cys affibody molecule was provided by Affibody AB (Solna, Sweden http://www.affibody.com) as a solution in 0.2 M sodium acetate. Phenyloxadiazolyl methylsulfone (PODS) was synthesized as described in the literature [15]. The maleimide-bearing affibody analogue NOTA-Z_EGFR:03115_ was prepared and radiolabeled as previously reported [22]. Low-protein-binding microcentrifuge tubes (1.5 mL) were purchased from Eppendorf (Stevenage, UK). Dry bifunctional chelators and affibody conjugates were achieved using a Concentrator Plus (Eppendorf, Stevenage, UK). Incubation of the reaction mixtures was performed using a Grant Bio thermo shaker (Camlab, Stevenage, UK). Protein conjugate concentration was determined by measuring the UV absorbance at 280 nm on a NanoDrop 2000 spectrophotometer (Thermo Scientific, Loughborough, UK) using a molar extinction coefficient (ε_280_) of 36345 cm^−1^ M^−1^. Oasis HLB (1 mL, 30 mg sorbent) SPE cartridges were purchased from Waters (Elstree, UK). [^18^F]Fluoride was produced a GE PETrace cyclotron by 16 MeV irradiation of an enriched [^18^O]H_2_O target, supplied by Alliance Medical Radiopharmarcy Ltd. (Warwick, UK) and used without further purification.

Analytical and semi-preparative HPLC were carried out on an Agilent Infinity 1260 quaternary pump system equipped with a 1260 Diode array (Agilent Technologies, Didcot, UK). Elution profiles were recorded using Laura 4 software (Lablogic, Sheffield, UK, 2013). NOTA-PODS and NODAGA-PODS were analyzed on an Eclipse XDB C18 column, 4.6 × 150 mm, 5 µm (Agilent Technologies, Didcot, UK) using Gradient 1: 0–20 min 3%–60% B, at flow rate of 1 mL/min with 0.1% TFA in water as eluent A and 0.1% TFA in acetonitrile as eluent B. The bifunctional chelators were purified by semi-preparative RP–HPLC on a Ultracarb ODS C18 column, 10 × 250 mm, 7 µm (Phenomenex, Macclesfield, UK), using Gradient 1a: 0–20 min 3%–60% B, at a flow rate of 3mL/min with 0.1% formic acid in water as eluent A and 0.1% formic acid in acetonitrile as eluent B and. Affibody conjugates and radioconjugates were analyzed on a Zorbax 300SB C18 column, 4.6 × 250 mm, 5 µm (Agilent Technologies, Didcot, UK) using Gradient 2: 0–20 min. 30%–65% B, with 0.1% TFA in water as eluent A and 0.1% TFA in acetonitrile as eluent B at a flow rate of 1 mL/min. Affibody conjugates were purified by semi-preparative RP–HPLC on a Jupiter C18 column, 10 × 250 mm, 10 µm, 300 Å (Phenomenex, Macclesfield, UK), using Gradient 2 at a 3 mL/min flow rate. The radioactivity of the eluate was monitored using an IN/US Systems Gamma-ram Model 4 NaI radiodetector (Lablogic, Sheffield, UK). Retention times (Rt) are expressed as minutes:seconds (min:sec). Electro spray ionization high-resolution mass spectrometry (ESI–HRMS) was performed using an Agilent 1200 series LC pump with a 6210 time-of-flight (TOF) mass analyzer. Protein MS was performed on 6520 a Series qToF mass spectrometer fitted with a dual ESI ionization source (Agilent, Santa Clara, CA, USA).

### 3.2. Preparation of NOTA-PODS and NODAGA-PODS

The macrocyclic chelator NHS ester (10.32 µmol), was added solid to a solution of PODS (6.88 µmol) in DMF (400 µL) and DIPEA (7.2 µL, 41.3 µmol). The clear solution was incubated in a thermo shaker at 23 °C for 2 h (900 rpm). The product was analyzed by RP–HPLC and either purified by RP–HPLC (Gradient 1a) or used for the next reaction without further purification.

NOTA-PODS = Analytical RP–HPLC (Gradient 1): Rt: 12:15 min:sec; ESI–HRMS: [M + H]^+^ (*m*/*z*) calcd: 827.9, found 827.3633.

NODAGA-PODS = Analytical RP–HPLC (Gradient 1): Rt: 12:13 min:sec; ESI–HRMS: [M + H]^+^ (*m*/*z*) calcd: 899.9, found 899.3895.

### 3.3. Preparation of NOTA-PODS-Z_EGFR:03115_ and NODA-PODS-Z_EGFR:03115_

In a 1.5 mL low-protein-binding centrifuge tube, a freshly prepared solution of TCEP-HCl in 1 M phosphate buffer pH 7 (0.025 mg/µL, 9.8 µL, 1.71 µmol) was added to a solution of Z_EGFR:03115_-Cys (200 µL, 2.3 mg/mL, 68.4 nmol). The mixture was incubated in a thermo shaker at 85 °C for 5 min (900 rpm) followed by 25 min at room temperature. Either NOTA-PODS or NODAGA-PODS solution in DMF was then added (1.03 µmol) and the solution was incubated in a thermo shaker at 37 °C for 1 h (900 rpm). The reaction mixture was analyzed by RP–HPLC. The product was purified by semi-preparative RP–HPLC. The collected fractions containing the product were dried and quantified by measuring the UV absorbance at 280 nm.

NOTA-PODS-Z_EGFR:03115_ = yield 38.5%. Analytical RP–HPLC (Gradient 2): Rt: 11:06 min:sec; ESI–HRMS (*m*/*z*): [M + H]^+^ expected: 7470, found: 7470.91.

NODAGA-PODS-Z_EGFR:03115_ = yield 39.9%. Analytical RP–HPLC (Gradient 2): Rt: 11:08 min:sec; ESI–HRMS (*m*/*z*): [M + H]^+^ expected: 7543, found: 7543.07.

### 3.4. Radiosynthesis of [^18^F]AlF-NOTA-PODS-Z_EGFR:03115_ and [^18^F]AlF-NODAGA-PODS-Z_EGFR:03115_

To a 1.5 mL low-protein-binding plastic tube containing either NOTA-PODS-Z_EGFR:03115_ or NODAGA-PODS-Z_EGFR:03115_ (lyophilized, 10–20 nmol), 2 mM AlCl_3_ (4.0–6.5 µL, 7–13 nmol) in 0.5 M sodium acetate buffer pH 4, 50 mM ascorbic acid in 25 mM sodium acetate pH 4.5 (to a final concentration of 1 mM), and aqueous non-purified [^18^F]fluoride (180–200 MBq) were added. A volume of ethanol was then added to achieve a final 1:1 *v*/*v* aqueous to organic solvent ratio. The mixture was heated at 100 °C for 15 min. After cooling to ambient temperature, the solution was purified by RP–HPLC using Gradient 2. The collected fraction containing the product was diluted with 0.1% aq TFA (3 mL) and loaded on an HLB-SPE cartridge (1 mL, 30 mg sorbent). The trapped radioactivity was washed with 0.1% aq TFA (4 mL) and then eluted with 60% ethanol/water (*v*/*v*, 120 µL). The product was quantified by measuring the UV absorbance at 280 nm. Synthesis time (from the beginning of the reaction) = ca. 45 min.

[^18^F]AlF-NOTA-PODS-Z_EGFR:03115_ = Analytical RP–HPLC (Gradient 2): Rt: 11:16 min:sec; RCY (decay corrected at the beginning of reaction): 11%–12.7%; apparent specific activity: 0.40–0.59 MBq/µg (apparent molar activity: 3.0–4.4 MBq/nmol).

[^18^F]AlF-NODAGA-PODS-Z_EGFR:03115_ = Analytical RP–HPLC (Gradient 2): Rt: 11:11 min:sec; RCY (decay corrected at the beginning of reaction): 4.3%–8.1%; apparent specific activity: 0.11–0.23 MBq/µg (apparent molar activity: 0.8–1.7 MBq/nmol).

### 3.5. Determination of Distribution Coefficient (LogD) at pH 7.4

To 0.5 mL of PBS (pH 7.4), either [^18^F]AlF-NOTA-PODS-Z_EGFR:03115_ or [^18^F]AlF-NODAGA-PODS-Z_EGFR:03115_ (0.08 MBq) was added followed by 0.5 mL of *n*-octanol. The mixture was vortexed for 10 min followed by centrifugation at 100× *g* for 10 min. The experiments were performed in triplicate. Three 100 µL samples were taken from each layer and the amount of activity was measured in a 2480 WIZARD^2^ Automatic Gamma Counter (Perkin Elmer, Beaconsfield, UK) as counts per minutes (cpm). The distribution coefficient at pH 7.4 (logD_7.4_) was expressed as the mean ± standard deviation (SD) and calculated using the following formula (Equation 1):

LogD = log[(counts_octanol_)/(counts_PBS_)] (1)

### 3.6. In Vitro Serum Stability Assay

The stability of [^18^F]AlF-NOTA-PODS-Z_EGFR:03115_ and [^18^F]AlF-NODAGA-PODS-Z_EGFR:03115_ was assessed as previously described by incubating the purified [^18^F]AlF-radioconjugates (3.5–4 MBq) in mouse serum (500 µL) in a thermo shaker at 37 °C for 1 h (850 rpm) [17,24]. Each experiment was performed in triplicate. The data are expressed as the average of n = 3 measurements ± SD.

### 3.7. In Vivo Evaluation

All experiments were performed in compliance with license issued under the UK Animals (Scientific Procedures) Act 1986 and following local ethical review (project license PCC916B22, Animals in Science Regulation Unit, Home Office Science, London, UK). The studies followed the United Kingdom National Cancer Research Institute Guidelines for Animal Welfare in Cancer Research [25]. Female NCr athymic mice (6–8 weeks old) were subcutaneously injected on the right shoulder with U87MGvIII cells (0.5 × 10^6^/mouse) suspended in 30% Matrigel. Tumors were allowed to grow to 100 mm^3^. PET/CT studies were conducted on an Albira PET/SPECT/CT imaging system (Bruker, Coventry, UK). Mice were administered the radioconjugate (12 µg in 100 µL of 0.9% sterile saline, 1.1–1.8 MBq/mouse) by intravenous tail vein injection and were anesthetized using an isoflurane/O_2_ mixture (1.5%–2.0% *v*/*v*) approximately 5 min prior to imaging. Whole-body static PET images were acquired 1 h post-radioconjugate injection for the duration of 10 min, with a 358 to 664 keV energy window, followed by CT acquisition as previously described [26]. The image data were processed and reconstructed as previously reported [26].

Immediately after image data acquisition, the mice were euthanized by cervical dislocation for the biodistribution studies. The major organs/tissues were dissected and weighed, and the radioactivity was measured in 2480 WIZARD^2^ Automatic Gamma Counter (Perkin Elmer, Beaconsfield, UK). The percentage of the injected dose per gram of tissue (%ID/g) was determined for each organ/tissue. The data are expressed as the average of *n* = 3 mice ± SD.

## 4. Conclusions

This study describes the preparation and attachment of two novel thiol-reactive PODS-bearing bifunctional chelators (NOTA-PODS and NODAGA-PODS) to the EGFR-targeting affibody molecule Z_EGFR:03115_. When radiolabeled with [^18^F]AlF, a RP–HPLC purification procedure followed by HLB-SPE was required to produce radioconjugates with a RCP > 98%. Overall, the radiolabeling efficiency for [^18^F]AlF-NODAGA-PODS-Z_EGFR:03115_ was found to be lower than for [^18^F]AlF-NOTA-PODS-Z_EGFR:03115_, a factor attributable to the chelator structure. Once purified, both radioconjugates showed a good serum stability. When injected in high EGFR-expressing tumor-bearing mice, [^18^F]AlF-NOTA-PODS-Z_EGFR:03115_ and [^18^F]AlF-NODAGA-PODS-Z_EGFR:03115_ showed similar pharmacokinetics and allowed for clear visualization of the tumors already 1 h post-injection. Additionally, the radiolabeling procedure, purification requirements, in vitro stability and in vivo behavior (at 1 h) of both PODS- and maleimide-bearing radioconjugates were found to be comparable. However, based on reports in the literature, it is possible that the benefits from the superior stability of PODS in vivo would be more noticeable at later time points. In conclusion, this investigation showed that PODS-based reagents are a viable alternative to maleimide for thiol-selective conjugation to cysteine-bearing proteins.

## Figures and Tables

**Table 1:** Summary of radiolabeling reactions. Radiochemical yields (RCY) and apparent specific (SA) and molar activities (MA) are decay corrected.

Radioconjugate	RCY	SA/MA	Protein Recovery
[^18^F]AIF-NOTA-PODS-Z_EGFR:03115_	11.0%–12.7%	0.40–0.59 MBq/μg 3.0–4.4 MBq/nmol	37.1%–38.0%
[^18^F]AIF-NODAGA-PODS-Z_EGFR:03115_	4.3%–8.1%	0.11–0.23 MBq/μg 0.8–1.7 MBq/nmol	23.9%–26.0%
[^18^F]AIF-NOTA-Z_EGFR:03115_	10.7%–38.0%	0.57–1.09 MBq/μg 4.1–7.8 MBq/nmol	10.6%–34.5%

**Table 2:** Tumor-to-organ ratios at 1 h p.i. of the three examined [^18^F]AlF conjugates. No differences were identified.

Tumor-to-Organ Ratio				
	Blood	Kidney	Liver	Muscle
[^18^F]AIF-NOTA-PODS-Z_EGFR:03115_	1.6 ± 0.6	0.2 ± 0.1	2.7 ± 1.2	37.2 ± 12.4
[^18^F]AIF-NODAGA-PODS-Z_EGFR:03115_	2.6 ± 1.2	0.1 ± 0.0	1.3 ± 0.6	31.3 ± 8.2
[^18^F]AIF-NOTA-Z_EGFR:03115_	2.8 ± 0.9	0.1 ± 0.0	4.7 ± 0.8	34.9 ± 3.4

## Supplementary Materials

The following are available online, Figure S1: RP–HPLC analysis (Gradient 1) of PODS (**A**), and the NODA-PODS (**B**) and NODAGA-PODS (**C**) reaction mixtures. NOTA-NHS and NODAGA-NHS elute with the mobile phase front, together with DMF (ca 3 min). The absorbance was recorded at the wavelength of 254 nm. The retention time (Rt) is indicated as min:sec. Figure S2: When the bifunctional chelators were purified by semi-preparative RPHPLC and subsequently dried using a speed-vacuum concentrator, the products showed clear signs of degradation RP–HPLC analysis (Gradient 1) of isolated NOTAPODS (**A**) and NODAGA-PODS (**B**). Each chromatogram shows the presence of one major degradation product (ca 10:40 min:sec). The absorbance was recorded at the wavelength of 254 nm. ESI–MS analysis of NOTA-PODS and the degradation product shows the expected mass of *m*/*z* 827 and a peak having a smaller mass (*m*/*z* 765) which could be associated to the hydrolysis derivative at the sulfone group (**C**). The prolonged presence of TFA in solution together with the type of drying process were possibly the cause. Figure S3: RP–HPLC analysis (Gradient 1) of pure NOTA-PODS (**A**) and NODAGAPODS (**B**) isolated by semi-preparative RP–HPLC using formic acid in the mobile phase instead of TFA. The absorbance was recorded at the wavelength of 254 nm. Figure S4: ESI–HRMS of NOTA-PODS (top) and NODAGA-PODS (bottom). Figure S5: RP–HPLC analysis of solutions of NOTA-PODS and NODAGA-PODS in DMF after being stored at −20 °C. Signs of degradation (peak at 10:36 min:sec) were detected already after 2 months for NOTA-PODS (**A**). Conversely, NODAGA-PODS showed good stability for at least 10 months (**B**). Figure S6: RP–HPLC analysis (Gradient 2) of NOTA-PODS-Z_EGFR:03115_ (A), and NODAGA-PODS-Z_EGFR:03115_ (**B**) reaction mixtures. NOTA-PODS and NODAGA-PODS elute with the mobile phase front, together with DMF (ca 3 min). The absorbance was recorded at the wavelength of 280 nm. The retention time (Rt) is indicated as min:sec. Figure S7: ESI–MS of purified NOTA-PODS-Z_EGFR:03115_ (top) and NODAGA-PODS-Z_EGFR:03115_ (bottom). Figure S8: Radiochromatograms (Gradient 2) of [^18^F]AlF-NOTA-PODS-Z_EGFR:03115_ (**A**), and [^18^F]AlF-NODAGA-PODS-Z_EGFR:03115_ (**B**) reaction mixtures. Free fluorine-18 elutes at ca 3 min. Labels on each peak on the chromatograms indicate the retention time (top) and the %ROI (bottom). Figure S9: Radiochromatograms (Gradient 2) of [^18^F]AlF-NOTA-PODS-Z_EGFR:03115_ (**A**), and [^18^F]AlF-NODAGA-PODS-Z_EGFR:03115_ (**B**) after purification by just HLB-SPE. As for [^18^F]AlF-NOTA-Z_EGFR:03115_ (**C**), the HLB-SPE-only purification step successfully removed the free fluorine-18 leaving the radioconjugate and the thermolysis products which elute at ca 3 min. The retention times (Rt) are expressed as min:sec. Figure S10: Representative radiochromatograms (Gradient 2) of [^18^F]AlF-NOTA-PODS-Z_EGFR:03115_ (**A**) and [^18^F]AlF-NODAGA-PODS-Z_EGFR:03115_ (**B**) after incubation in mouse serum for 1 h. The intact radioconjugates elute at ca 11 min. Activity non-associated with the conjugate elutes at ca 3 min. Labels on each peak on the chromatograms indicate the retention time (top) and the %ROI (bottom). Table S1: Summary of LogD_7.4_ values measured for the three radioconjugates. Table S2: Summary of serum stability determined by RP–HPLC. The three radioconjugates were incubated in mouse serum at 37 °C for 1 h. The data are shown as the mean values of *n* = 3 experiments ± SD. Statistical analysis was performed using one-way ANOVA with Tukey correction using GraphPad Prism v8. Table S3: Summary of serum stability determined by RP–HPLC. The three radioconjugates were incubated in mouse serum at 37 °C for 1 h. The data are shown as the mean values of *n* = 3 experiments ± SD. Statistical analysis was performed using one-way ANOVA with Tukey correction using GraphPad Prism v8.

## References

[1] Gunnoo S.B., Madder A. (2016). Chemical protein modification through cysteine. ChemBioChem.

[2] Koniev O., Wagner A. (2015). Developments and recent advancements in the field of endogenous amino acid selective bond forming reactions for bioconjugation. Chem. Soc. Rev..

[3] Ravasco J.M.J.M., Faustino H., Trindade A., Gois P.M.P. (2019). Bioconjugation with maleimides: A useful tool for chemical biology. Chem. Eur. J..

[4] Sabot C., Renard P.-Y., Renault K., Fredy J.W. (2018). Covalent modification of biomolecules through maleimide-based labeling strategies. Bioconjugate Chem..

[5] Alley S.C., Benjamin D.R., Jeffrey S.C., Okeley N.M., Meyer D.L., Sanderson R.J., Senter P.D. (2008). Contribution of linker stability to the activities of anticancer immunoconjugates. Bioconjugate Chem..

[6] Baldwin A.D., Kiick K.L. (2011). Tunable degradation of maleimide–thiol adducts in reducing environments. Bioconjugate Chem..

[7] Ponte J.F., Sun X., Yoder N.C., Fishkin N., Laleau R., Coccia J., Lanieri L., Bogalhas M., Wang L., Wilhelm S. (2016). Understanding how the stability of the thiol-maleimide linkage impacts the pharmacokinetics of lysine-linked antibody-maytansinoid conjugates. Bioconjugate Chem..

[8] Szijj P.A., Bahou C., Chudasama V. (2018). Minireview: Addressing the retro-Michael instability of maleimide bioconjugates. Drug Discov. Today Technol..

[9] Bernardim B., Cal P.M.S.D., Matos M.J., Oliveira B.L., Martínez-Sáez N., AlbuquerqueInês S., Perkins E., Corzana F., Burtoloso A.C.B., Jiménez-Osés G. (2016). Stoichiometric and irreversible cysteine-selective protein modification using carbonylacrylic reagents. Nat. Commun..

[10] Kalia D., Malekar P.V., Parthasarathy M. (2016). Exocyclic olefinic maleimides: Synthesis and application for stable and thiol-selective bioconjugation. Angew. Chem. Int. Ed. Engl..

[11] Toda N., Asano S., Barbas C.F.I. (2013). Rapid, stable, chemoselective labeling of thiols with Julia-Kocieński-like reagents: A serum-stable alternative to maleimide-based protein conjugation. Angew. Chem. Int. Ed. Engl..

[12] Patterson J.T., Asano S., Li X., Rader C., Barbas C.F.I. (2014). Improving the serum stability of site-specific antibody conjugates with sulfone linkers. Bioconjugate Chem..

[13] Zhang Q., Dall’Angelo S., Fleming I.N., Schweiger L.F., Zanda M., O’Hagan D. (2016). Last-step enzymatic [^18^F]-fluorination of cysteine-tethered RGD peptides using modified Barbas linkers. Chem. Eur. J..

[14] Chiotellis A., Sladojevich F., Mu L., Müller Herde A., Valverde I.E., Tolmachev V., Schibli R., Ametamey S.M., Mindt T.L. (2016). Novel chemoselective ^18^F-radiolabeling of thiol-containing biomolecules under mild aqueous conditions. Chem. Commun..

[15] Adumeau P., Davydova M., Zeglis B.M. (2018). Thiol-reactive bifunctional chelators for the creation of site-selectively modified radioimmunoconjugates with improved stability. Bioconjug. Chem..

[16] McBride W.J., Sharkey R.M., Karacay H., D’Souza C.A., Rossi E.A., Laverman P., Chang C.H., Boerman O.C., Goldenberg D.M. (2009). A novel method of 18F radiolabeling for PET. J. Nucl. Med..

[17] Da Pieve C., Allott L., Martins C.D., Vardon A., Ciobota D.M., Kramer-Marek G., Smith G. (2016). Efficient [^18^F]AlF radiolabeling of Z_HER3:8698_ affibody molecule for imaging of HER3 positive tumors. Bioconjug. Chem..

[18] Kim Y., Ho S.O., Gassman N.R., Korlann Y., Landorf E.V., Collart F.R., Weiss S. (2008). Efficient site-specific labeling of proteins via cysteines. Bioconjug. Chem..

[19] Liu Y., Hu X., Liu H., Bu L., Ma X., Cheng K., Li J., Tian M., Zhang H., Cheng Z. (2013). A comparative study of radiolabeled bombesin analogs for the PET imaging of prostate cancer. J. Nucl. Med..

[20] D’Souza C.A., McBride W.J., Sharkey R.M., Todaro L.J., Goldenberg D.M. (2011). High-yielding aqueous ^18^F-labeling of peptides via Al^18^F chelation. Bioconjug. Chem..

[21] Löfblom J., Feldwisch J., Tolmachev V., Carlsson J., Ståhl S., Frejd F.Y. (2010). Affibody molecules: Engineered proteins for therapeutic, diagnostic and biotechnological applications. Febs Lett..

[22] Burley T.A., Da Pieve C., Martins C.D., Ciobota D.M., Allott L., Oyen W.J.G., Harrington K.J., Smith G., Kramer-Marek G. (2019). Affibody-Based PET Imaging to Guide EGFR-Targeted Cancer Therapy in Head and Neck Squamous Cell Cancer Models. J. Nucl. Med..

[23] Behr T.M., Goldenberg D.M., Becker W. (1998). Reducing the renal uptake of radiolabeled antibody fragments and peptides for diagnosis and therapy: Present status, future prospects and limitations. Eur. J. Nucl. Med..

[24] Su X., Cheng K., Jeon J., Shen B., Venturin G.T., Hu X., Rao J., Chin F.T., Wu H., Cheng Z. (2014). Comparison of two site-specifically ^18^F-labeled affibodies for PET imaging of EGFR positive tumors. Mol. Pharm..

[25] Workman P., Aboagye E.O., Balkwill F., Balmain A., Bruder G., Chaplin D.J., Double J.A., Everitt J., Farningham D.A.H., Glennie M.J. (2010). Guidelines for the welfare and use of animals in cancer research. Br. J. Cancer.

[26] Martins C.D., Da Pieve C., Burley T.A., Smith R., Ciobota D.M., Allott L., Harrington K.J., Oyen W.J.G., Smith G., Kramer-Marek G. (2018). HER3-mediated resistance to Hsp90 inhibition detected in breast cancer xenografts by affibody-based PET imaging. Clin. Cancer Res..

