# Supplementary material for: Thiol-Reactive PODS-Bearing Bifunctional Chelators for the Development of EGFR-Targeting [18F]AlF-Affibody Conjugates
Source: Molecules. 2020 Mar 29;25(7):1562. doi: 10.3390/molecules25071562 (PMC7180749; doi:10.3390/molecules25071562)
Supplement: Supplementary file 1 [file molecules-25-01562-s001.pdf]

Supporting Information

Thiol-reactive PODS-bearing bifunctional chelators for the development of EGFR targeting [^{18}F]AlF-affibody conjugates

Chiara Da Pieve[1*], Ata Makarem[2], Stephen Turnock[3], Justyna Maczynska[3], Graham Smith[1], Gabriela Kramer-Marek[3*]

[1] PET Radiochemistry, Division of Radiotherapy and Imaging, The Institute of Cancer Research, 123 Old Brompton Road, London, SW7 3RP, UK

[2] Division of Radiopharmaceutical Chemistry, German Cancer Research Center (DKFZ), Im Neuenheimer Feld 280, 69120 Heidelberg, Germany

[3] Preclinical Molecular Imaging, Division of Radiotherapy and Imaging, The Institute of Cancer Research, 123 Old Brompton Road, London, SW7 3RP, UK

Contents

Preparation and stability of bifunctional chelators NOTA-PODS and NODAGA-PODS page 2

Preparation of NOTA-PODS-$Z_{EGFR:03115}$ and NODAGA-PODS-$Z_{EGFR:03115}$ page 7

[^{18}F]AlF radiolabelling and *in vitro* stability page 9

In vivo studies page 13

Preparation and stability of bifunctional chelators NOTA-PODS and NODAGA-PODS

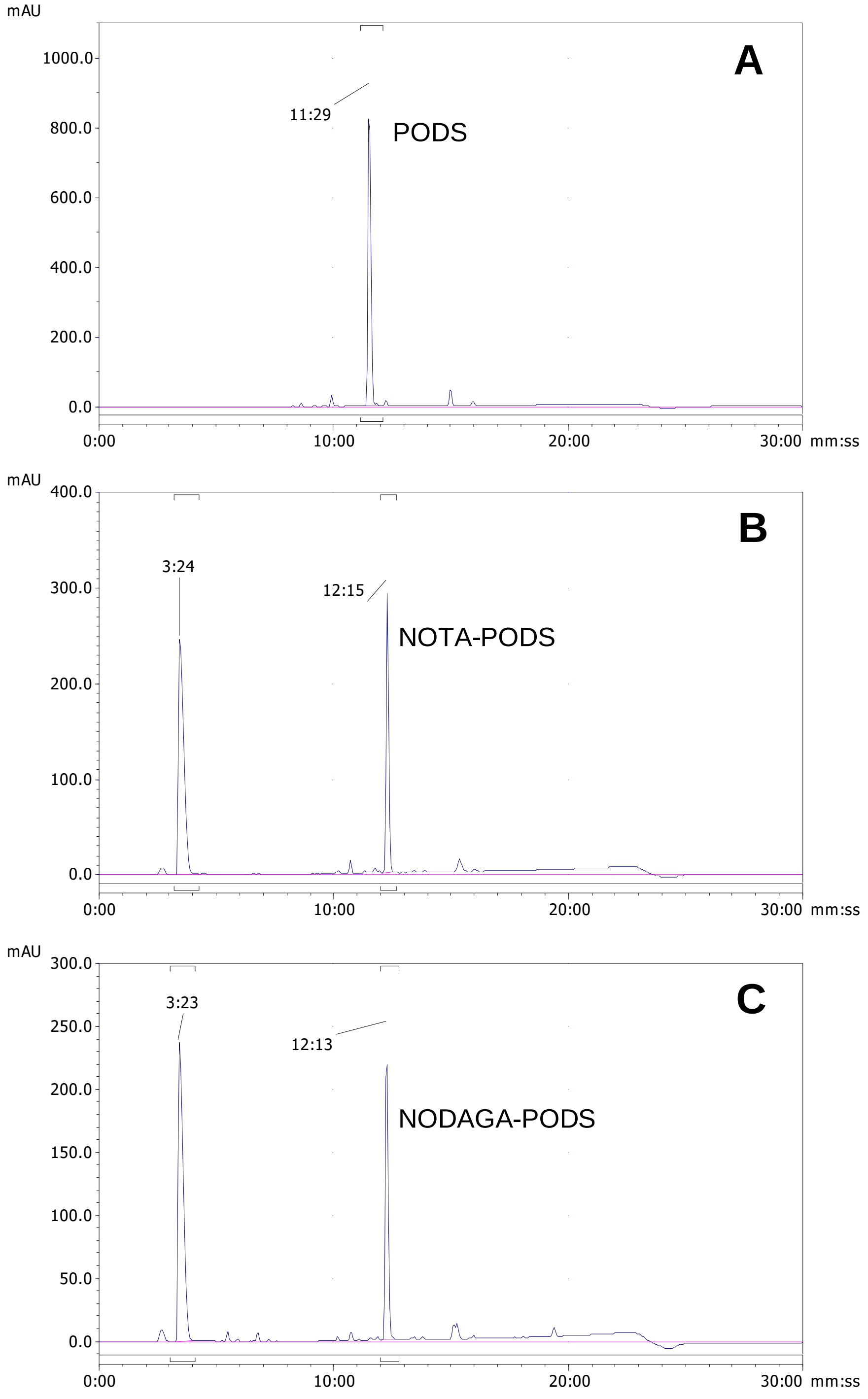

Figure S1. RP-HPLC analysis (Gradient 1) of PODS (A), and the NODA-PODS (B) and NODAGA-PODS (C) reaction mixtures. NOTA-NHS and NODAGA-NHS elute with the mobile phase front, together with DMF (ca 3 min.). The absorbance was recorded at the wavelength of 254 nm. The retention time (Rt) is indicated as min:sec.

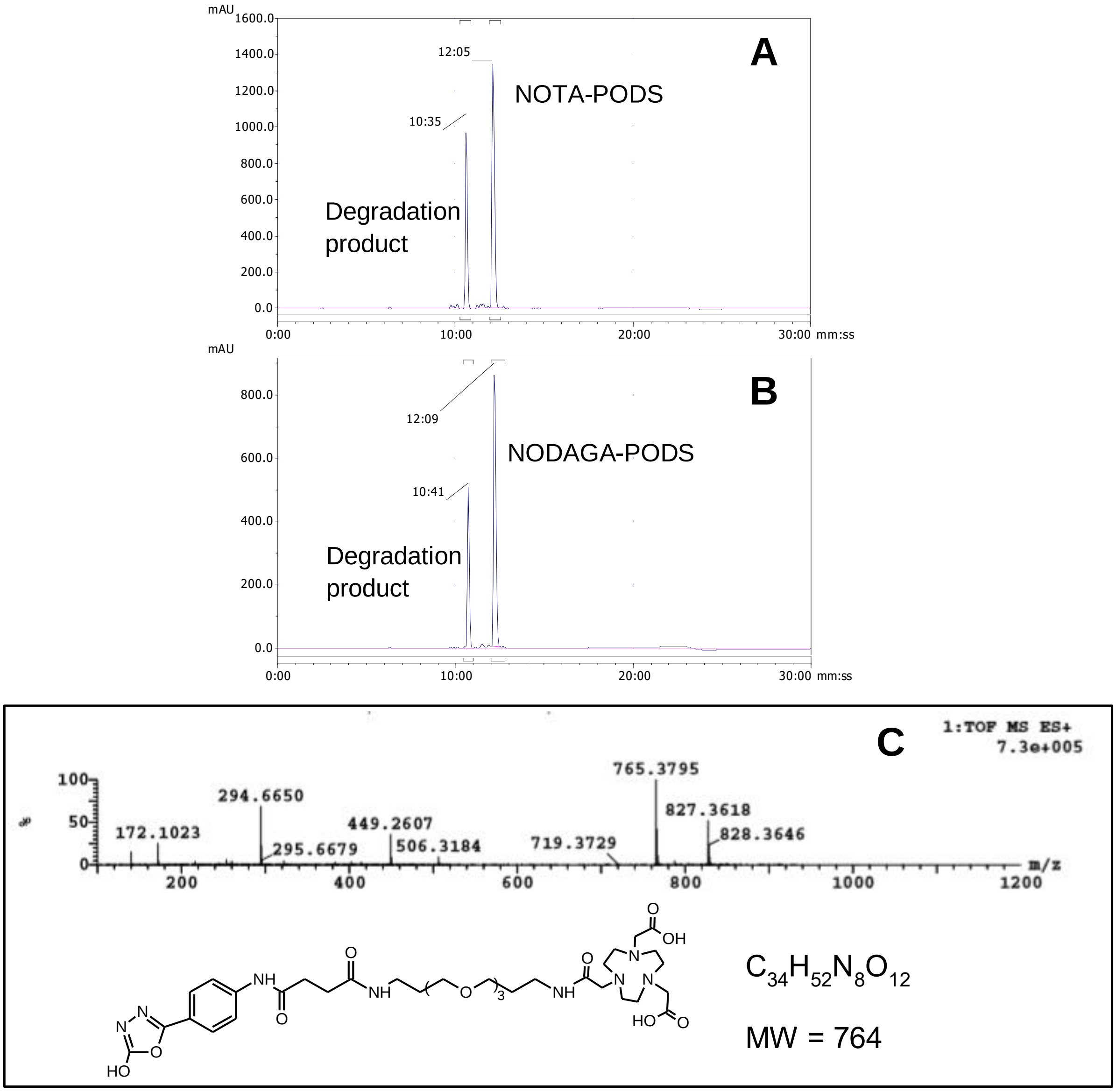

Figure S2. When the bifunctional chelators were purified by semi-preparative RP-HPLC and subsequently dried using a speed-vacuum concentrator, the products showed clear signs of degradation RP-HPLC analysis (Gradient 1) of isolated NOTA-PODS (A) and NODAGA-PODS (B). Each chromatograms show the presence of one major degradation product (ca 10:40 min:sec). The absorbance was recorded at the wavelength of 254 nm. ESI-MS analysis of NOTA-PODS and the degradation product shows the expected mass of m/z 827 and a peak having a smaller mass (m/z 765) which could be associated to the hydrolysis derivative at the sulfone group (C). The prolonged presence of TFA in solution together with the type of drying process were possibly the cause.

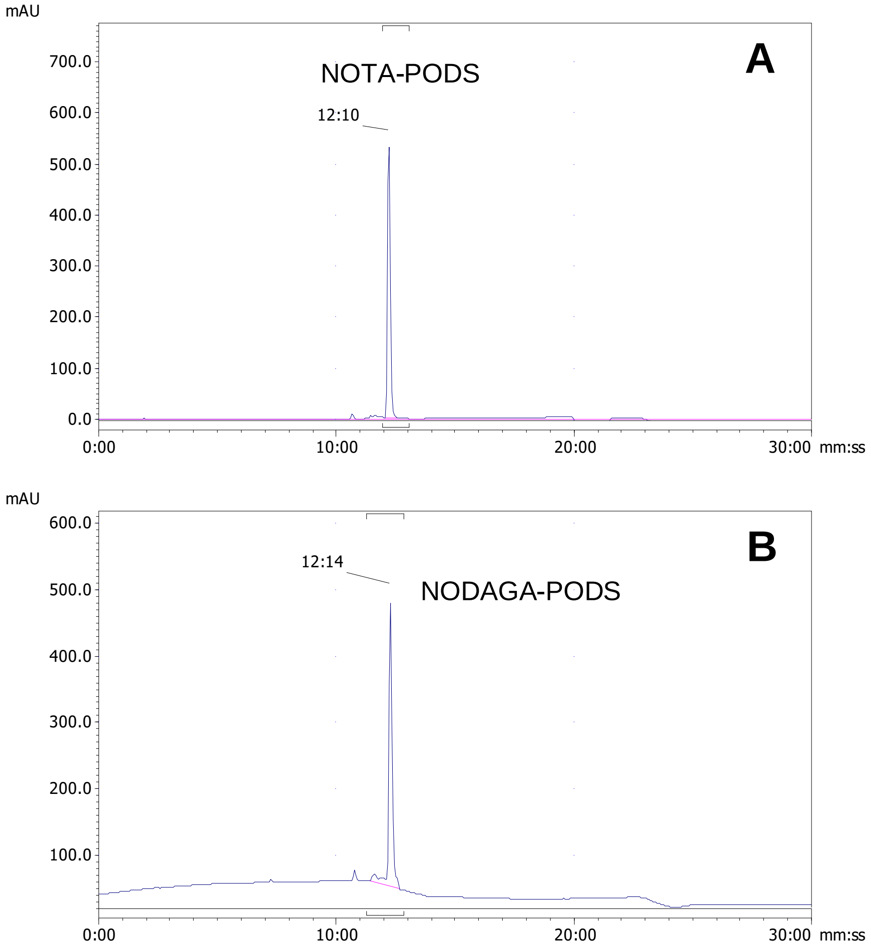

Figure S3. RP-HPLC analysis (Gradient 1) of pure NOTA-PODS (A) and NODAGA-PODS (B) isolated by semi-preparative RP-HPLC using formic acid in the mobile phase instead of TFA. The absorbance was recorded at the wavelength of 254 nm.

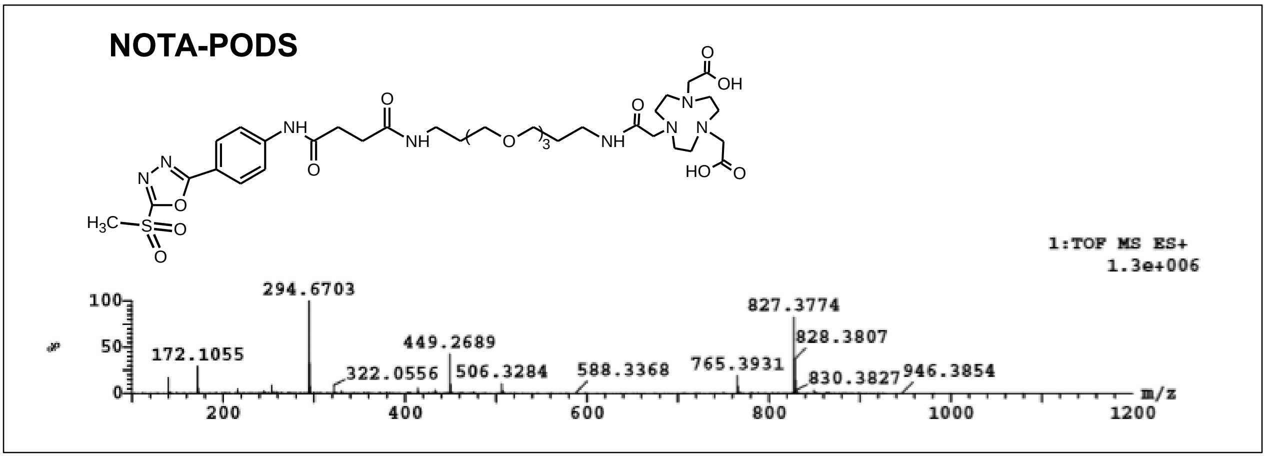

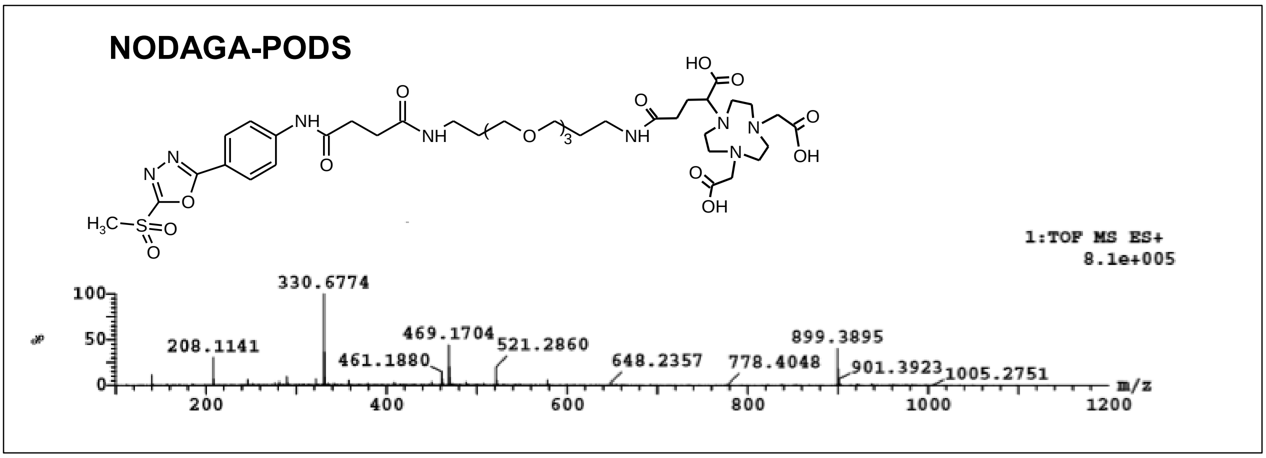

Figure S4. ESI-HRMS of NOTA-PODS (top) and NODAGA-PODS (bottom).

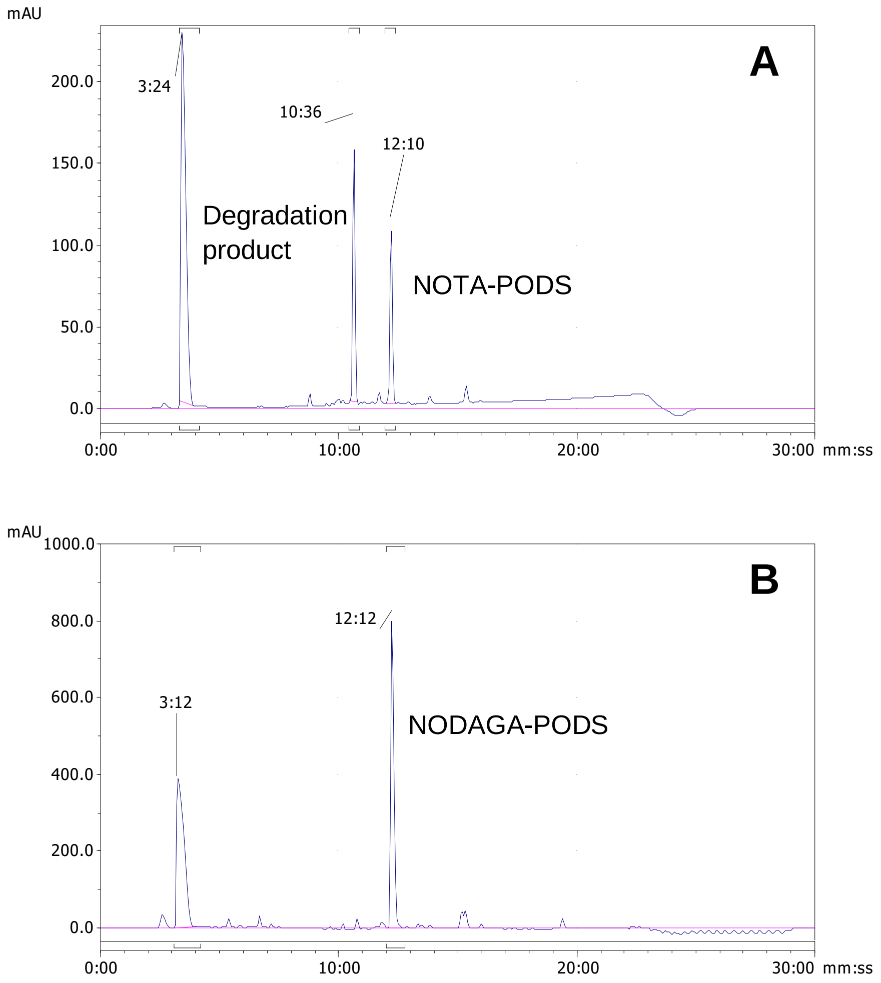

Figure S5. RP-HPLC analysis of solutions of NOTA-PODS and NODAGA-PODS in DMF after being stored at -20ºC. Signs of degradation (peak at 10:36 min:sec) were detected already after 2 months for NOTA-PODS (A). Conversely, NODAGA-PODS showed good stability for at least 10 months (B).

Preparation of NOTA-PODS-$Z_{EGFR:03115}$ and NODAGA-PODS-$Z_{EGFR:03115}$

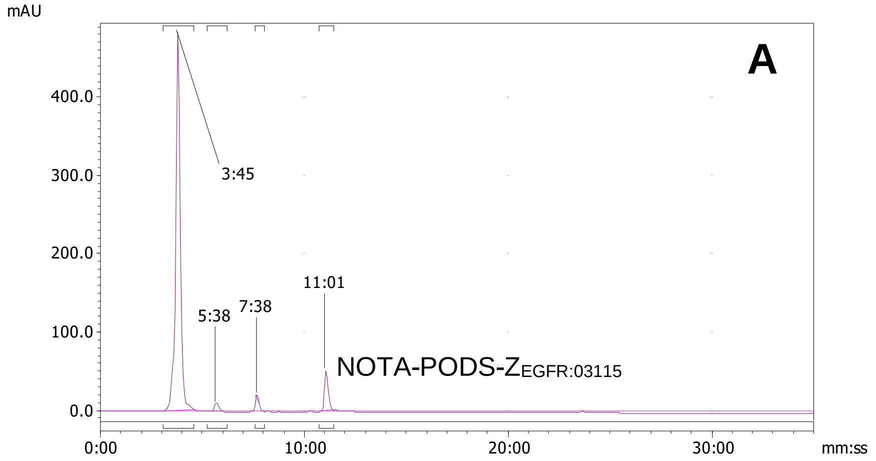

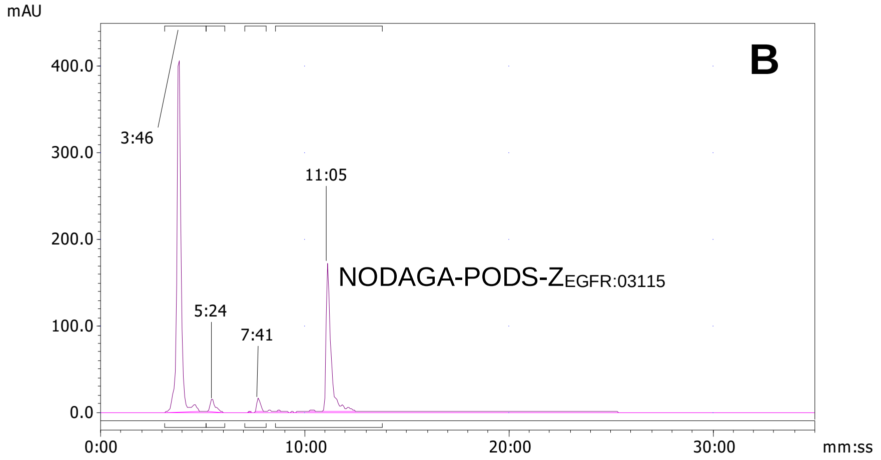

Figure S6. RP-HPLC analysis (Gradient 2) of NOTA-PODS-$Z_{EGFR:03115}$ (A), and NODAGA-PODS-$Z_{EGFR:03115}$ (B) reaction mixtures. NOTA-PODS and NODAGA-PODS elute with the mobile phase front, together with DMF (ca 3 min.). The absorbance was recorded at the wavelength of 280 nm. The retention time (Rt) is indicated as min:sec.

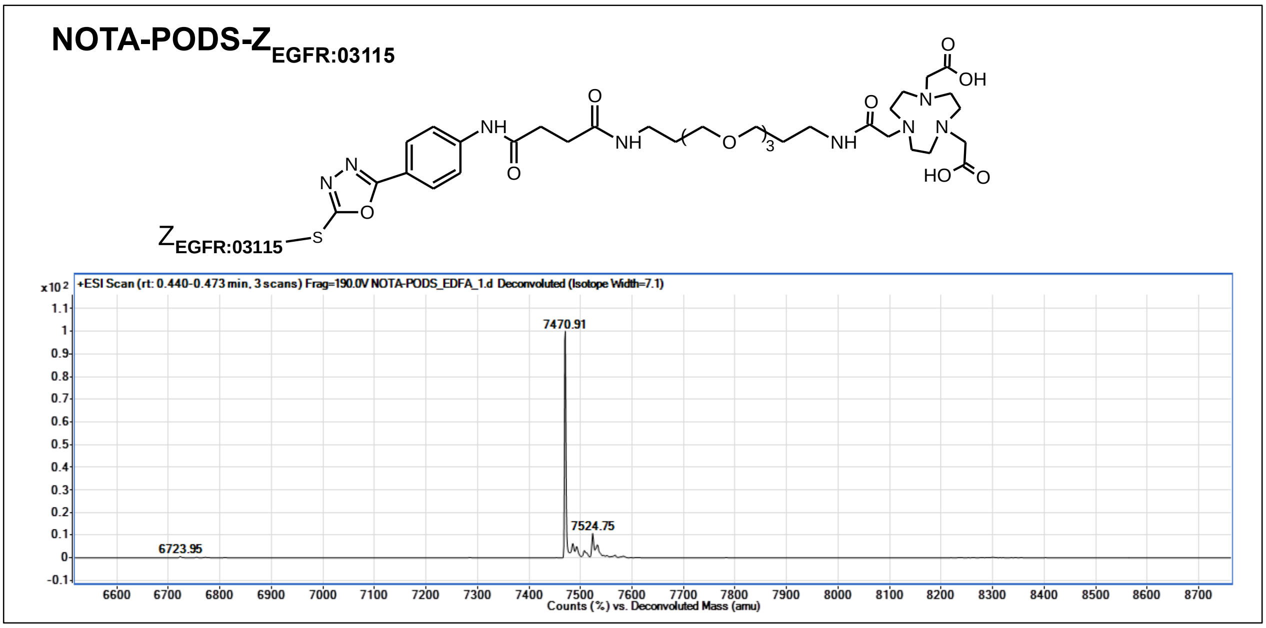

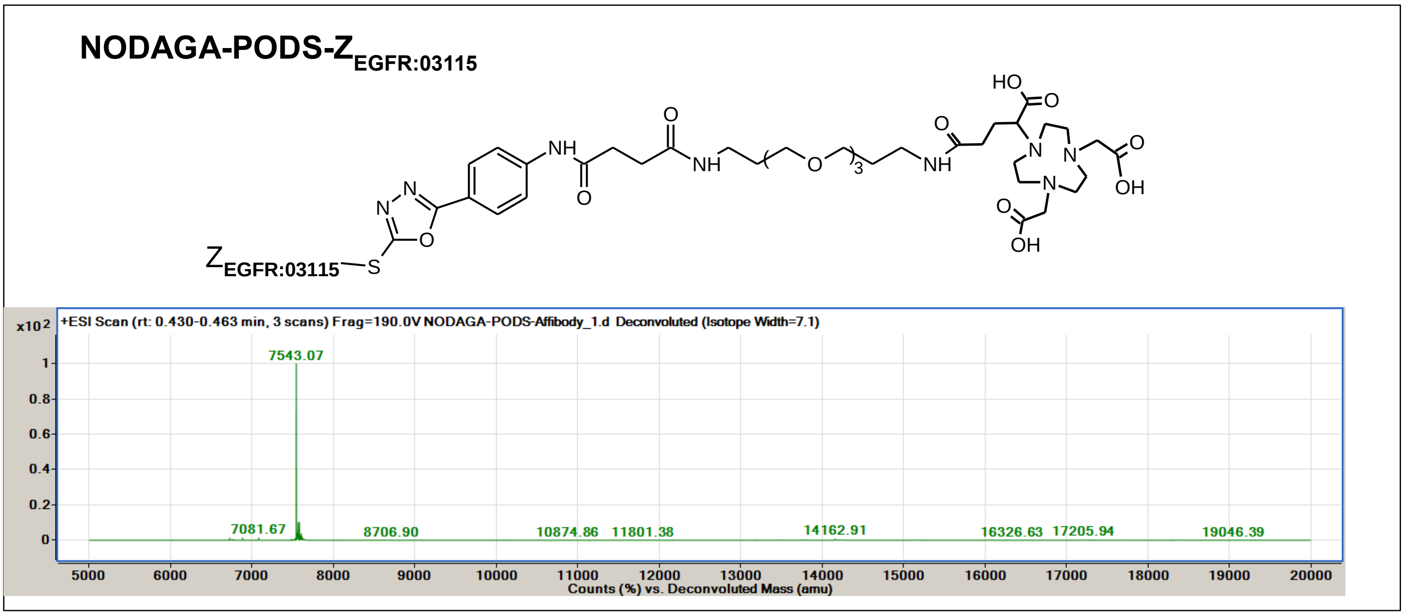

Figure S7. ESI-MS of purified NOTA-PODS-$Z_{EGFR:03115}$ (top) and NODAGA-PODS-$Z_{EGFR:03115}$ (bottom).

[^{18}F]AlF radiolabelling and *in vitro* stability

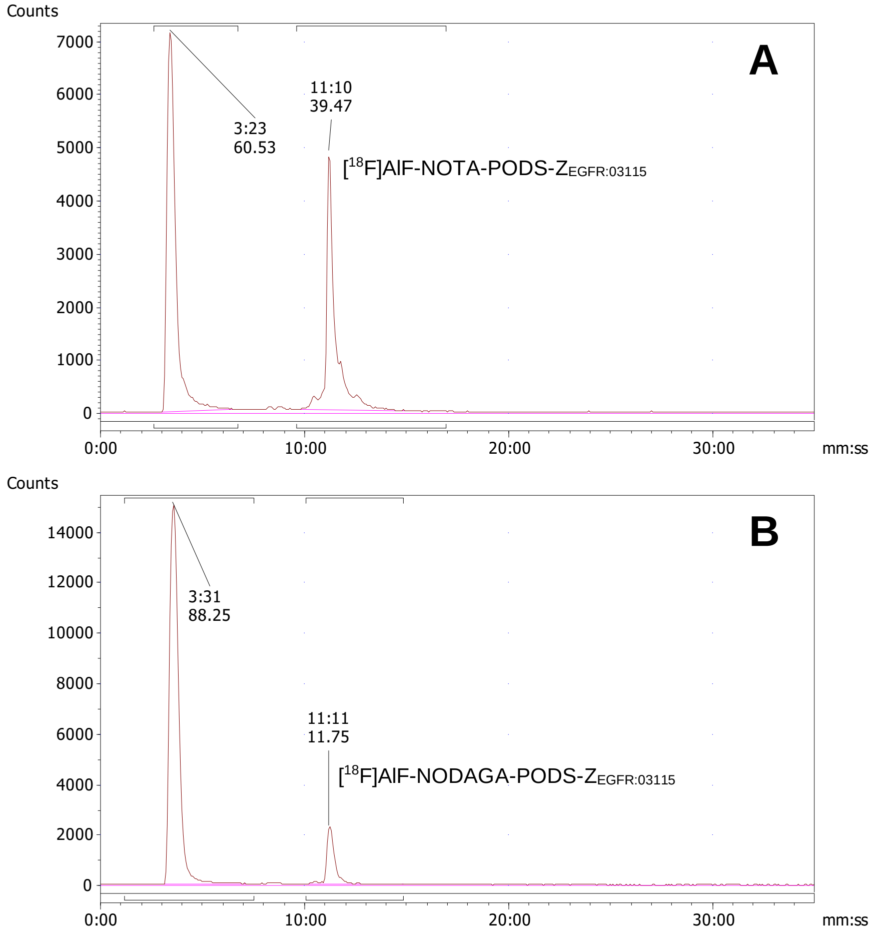

Figure S8. Radiochromatograms (Gradient 2) of [^{18}F]AlF-NOTA-PODS-$Z_{EGFR:03115}$ (A), and [^{18}F]AlF-NODAGA-PODS-$Z_{EGFR:03115}$ (B) reaction mixtures. Free fluorine-18 elutes at ca 3 min. Labels on each peak on the chromatograms indicate the retention time (top) and the %ROI (bottom).

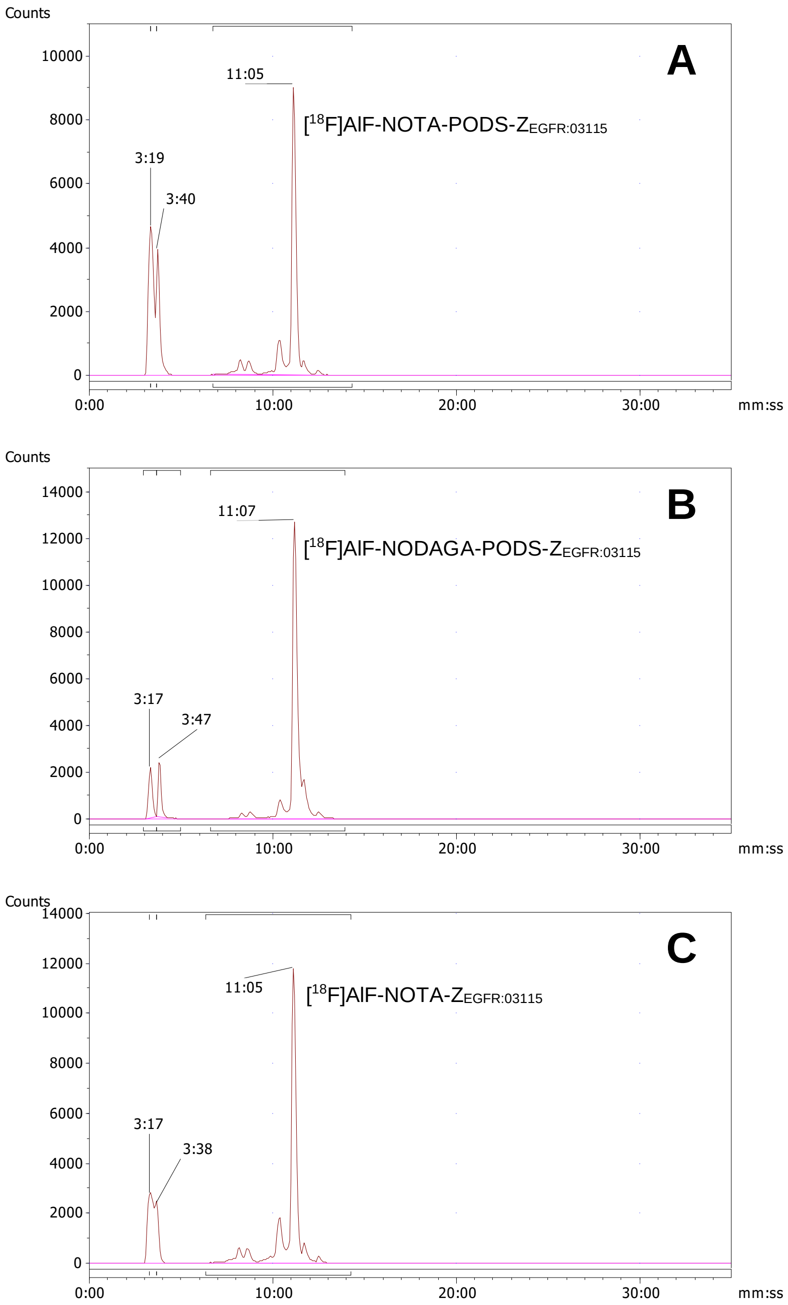

Figure S9. Radiochromatograms (Gradient 2) of [^{18}F]AlF-NOTA-PODS-Z$_{EGFR:03115}$ (A), and [^{18}F]AlF-NODAGA-PODS-Z$_{EGFR:03115}$ (B) after purification by just HLB-SPE. As for [^{18}F]AlF-NOTA-Z$_{EGFR:03115}$ (C), the HLB-SPE-only purification step successfully removed the free fluorine-18 leaving the radioconjugate and the thermolysis products which elute at ca 3 min. The retention times (Rt) are expressed as min:sec.

Radioconjugate	$LogD_{7.4}$
**[^{18}F]AlF-NOTA-PODS-$Z_{EGFR:03315}$**	-1.73 ±0.07
**[^{18}F]AlF-NODAGA-PODS-$Z_{EGFR:03115}$**	-3.62 ±0.06
**[^{18}F]AlF-NOTA-$Z_{EGFR:03115}$**	-1.13 ±0.1

Table S1. Summary of $LogD_{7.4}$ values measured for the three radioconjugates.

Radioconjugate	Fraction of intact radioconjugate (% ±SD)	Protein-associated activity (% ±SD)
**[^{18}F]AlF-NOTA-PODS-$Z_{EGFR:03315}$**	92.7 ± 2.6	24.2 ±3.4[a]
**[^{18}F]AlF-NODAGA-PODS-$Z_{EGFR:03115}$**	97.2 ± 1.2	20.4 ±0.7[b]
**[^{18}F]AlF-NOTA-$Z_{EGFR:03115}$**	95.8 ± 0.7	30.5 ± 2.1

[a] Significantly lower value ($P < 0.01$) compared to [^{18}F]AlF-NOTA-$Z_{EGFR:03115}$

[b] Significantly lower value ($P < 0.001$) compared to [^{18}F]AlF-NOTA-$Z_{EGFR:03115}$

Table S2. Summary of serum stability determined by RP-HPLC. The three radioconjugates were incubated in mouse serum at 37°C for 1 h. The data are shown as the mean values of n = 3 experiments ± SD. Statistical analysis was performed using one-way ANOVA with Tukey correction using GraphPad Prism v8.

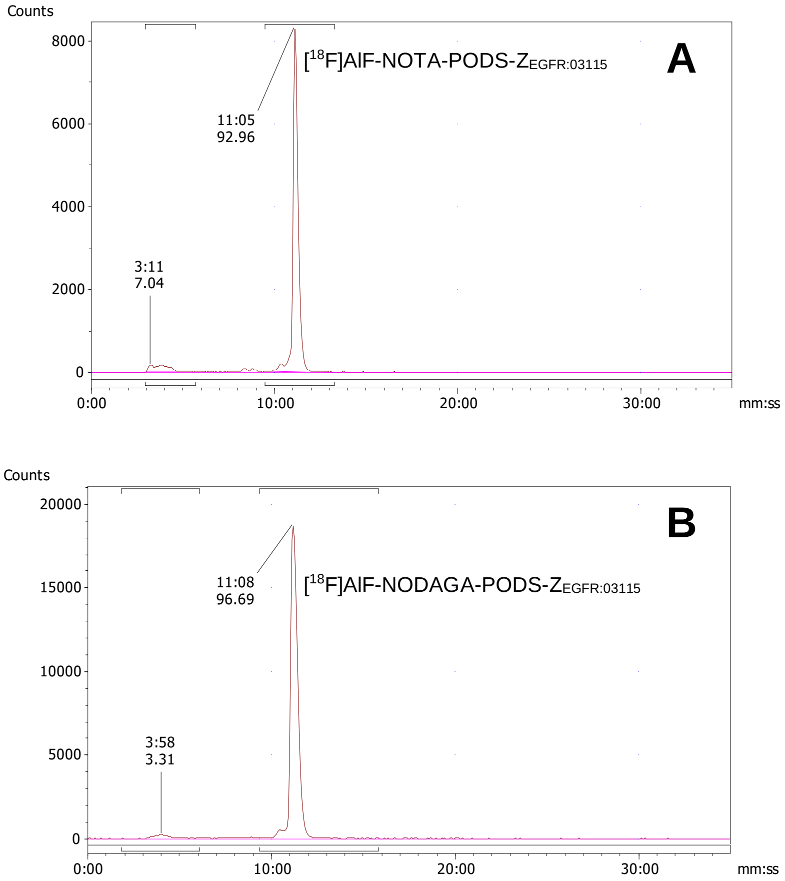

Figure S10. Representative radiochromatograms (Gradient 2) of [^{18}F]AlF-NOTA-PODS-$Z_{EGFR:03115}$ (A) and [^{18}F]AlF-NODAGA-PODS-$Z_{EGFR:03115}$ (B) after incubation in mouse serum for 1 h. The intact radioconjugates elute at ca 11 min. Activity non-associated with the conjugate elutes at ca 3 min. Labels on each peak on the chromatograms indicate the retention time (top) and the %ROI (bottom).

In vivo studies

Organ	[18F]AlF-NOTA-PODS-$Z_{EGFR:03115}$	[18F]AlF-NODAGA-PODS-$Z_{EGFR:03115}$	[18F]AlF-NOTA-$Z_{EGFR:03115}$
**Blood**	8.7 ±1.4	7.6 ±4.0	7.8 ±1.5
**Heart**	1.8 ±0.4	1.8 ±0.8	1.7 ±0.3
**Lungs**	3.5 ±0.4	3.8 ±1.6	4.5 ±0.5
**Kidney**	78.5 ±9.2	125.1 ±10.3[a]	145.0 ±17.4[a,b]
**Spleen**	1.1 ±0.2	1.9 ±0.3	1.5 ±0.2
**Liver**	5.3 ±1.2	14.6 ±7.8	4.3 ±0.7
**Pancreas**	0.9 ±0.03	1.5 ±0.5	1.1 ±0.1
**Tumor**	14.1 ±5.3	16.7 ±4.5	20.2 ±2.9
**Bone**	1.0 ±0.1	1.0 ±0.7	1.3 ±0.2
**Small Intestine**	1.2 ±0.2	2.1 ±0.9	2.1 ±0.2
**Muscle**	0.4 ±0.03	0.5 ±0.2	0.6 ±0.04
**Brain**	0.1 ±0.02	0.2 ±0.1	0.2 ±0.04
**Stomach**	4.3 ±1.7	3.2 ±0.6	3.2 ±0.7

[a] Significantly higher uptake ($P < 0.001$) compared to [18F]AlF-NOTA-PODS-$Z_{EGFR:03115}$

[b] Significantly higher uptake ($P < 0.001$) compared to [18F]AlF-NODAGA-PODS-$Z_{EGFR:03115}$

Table S3. Biodistribution results for [18F]AlF-NOTA-PODS-$Z_{EGFR:03115}$ and [18F]AlF-NODAGA-PODS-$Z_{EGFR:03115}$ (1.1-1.8 MBq/mouse) at 1 h p.i. [18F]AlF-NOTA-$Z_{EGFR:03115}$ was used as a comparison. The data are reported as the mean percentage of the injected dose per gram of tissue (%ID/g) ± SD (for each group, n = 3). Statistical analysis was performed using a two-way ANOVA with Tukey correction using GraphPad Prism v8.
